# Positive Selection Drives the Adaptive Evolution of Mitochondrial Antiviral Signaling (MAVS) Proteins-Mediating Innate Immunity in Mammals

**DOI:** 10.3389/fvets.2021.814765

**Published:** 2022-01-31

**Authors:** Hafiz Ishfaq Ahmad, Gulnaz Afzal, Muhammad Nouman Iqbal, Muhammad Arslan Iqbal, Borhan Shokrollahi, Muhammad Khalid Mansoor, Jinping Chen

**Affiliations:** ^1^Department of Animal Breeding and Genetics, University of Veterinary and Animal Sciences, Lahore, Pakistan; ^2^Department of Zoology, The Islamia University of Bahawalpur, Bahawalpur, Pakistan; ^3^Rural Health Centre Sardar Pur Kabirwala, Khanewal, Pakistan; ^4^District Head Quarter Hospital, Muzaffargarh, Pakistan; ^5^Department of Animal Science, Sanandaj Branch, Islamic Azad University, Sanandaj, Iran; ^6^Department of Microbiology, Faculty of Veterinary and Animal Science, The Islamia University of Bahawalpur, Bahawalpur, Pakistan; ^7^Guangdong Key Laboratory of Animal Conservation and Resource Utilization, Guangdong Public Laboratory of Wild Animal Conservation and Utilization, Institute of Zoology, Guangdong Academy of Sciences, Guangzhou, China

**Keywords:** MAVS, mammals, innate immunity, adaptive evolution, mitochondria, positive selection

## Abstract

The regulated production of filamentous protein complexes is essential in many biological processes and provides a new paradigm in signal transmission. The mitochondrial antiviral signaling protein (MAVS) is a critical signaling hub in innate immunity that is activated when a receptor induces a shift in the globular caspase activation and recruitment domain of MAVS into helical superstructures (filaments). It is of interest whether adaptive evolution affects the proteins involved in innate immunity. Here, we explore and confer the role of selection and diversification on mitochondrial antiviral signaling protein in mammalian species. We obtined the MAVS proteins of mammalian species and examined their differences in evolutionary patterns. We discovered evidence for these proteins being subjected to substantial positive selection. We demonstrate that immune system proteins, particularly those encoding recognition proteins, develop under positive selection using codon-based probability methods. Positively chosen regions within recognition proteins cluster in domains involved in microorganism recognition, implying that molecular interactions between hosts and pathogens may promote adaptive evolution in the mammalian immune systems. These significant variations in MAVS development in mammalian species highlights the involvement of MAVS in innate immunity. Our findings highlight the significance of accounting for how non-synonymous alterations affect structure and function when employing sequence-level studies to determine and quantify positive selection.

## Introduction

Immune responses in mammalian cells are dependent on the exposure to conserved molecular patterns present in pathogens, such as bacterial flagellins, lipoproteins, peptidoglycans, and lipopolysaccharides, as well as viral nucleic acids (1). Identification of pathogen-derived nucleic acid is an important process of the host defense cells against invading pathogens. After identifying foreign invaders, the transcription of antiviral genes results in the cellular antiviral state that arms the cells to battle and subdues the infection. The host cells' sensors against pathogen-associated DNA and RNA exist (2). Therefore, mitochondria are important sensors in antiviral immunity through their key role in apoptosis (3). One of the most important defense mechanisms against viral infections is the removal of infected cells through apoptosis. Mitochondria are chief performers in antiviral immunity because of their key role in apoptosis (3).

A new mitochondrial protein called mitochondrial antiviral signaling protein (MAVS), along with mitochondrial DNA acting as a danger-associated molecular pattern (DAMP) and mitochondrial ROS generated from mitochondrial sources, may establish mitochondria as key signaling platforms in antiviral immunity in vertebrates (4). In recent years, a thorough understanding of the emerging and intervening role of mitochondria in toll-like receptor-mediated innate immune responses and the activation of the NLRP3 inflammasome complex has gained clarity, which supports the idea that mitochondria have imposing functions in the context of innate immunity (5).

The identification of Retinoic acid Inducible Gene I-(RIG-I)-Like Receptors (RLRs), which are RNA sensing cytosolic receptors that require mitochondrial antiviral signaling protein adaptors to activate the production of interferons and pro-inflammatory cytokines against invading pathogens, revealed the role of mitochondria in innate antiviral immunity (6). RLRs are a set of germline-encoded Pattern Recognition Receptors (PRRs) that directly activate immune cells against invading organisms (7). Previous research has demonstrated that non-microbial danger signals, also known as danger-associated molecular patterns, are composed of host chemicals released by necrotic cells in response to tissue damage. These signals activate innate immune response (8). One of the most exciting discoveries in the last decade about the specific roles of mitochondria has been their role in cellular innate antiviral immunity in vertebrates, particularly mammals (9, 10). MAVS is located on the outer membrane of the mitochondria and has been associated with peroxisomes, the endoplasmic reticulum, and autophagosomes, where it coordinates signaling processes downstream of RLRs. MAVS has a role in regulating apoptotic and metabolic activities and activating antiviral and inflammatory pathways. The MAVS adaptor and its important role in the innate immune response to RNA viruses are highlighted in this study (11).

According to recent research, activation of MAVS molecules results in the polymerization of the molecules, which results in the formation of functional groups (12). As with prion fibers, these high-molecular-weight aggregates have properties comparable to those of prion fibers. They are resistant to detergent or protease and may self-replicate by encouraging inactive protein to produce functional groups. The CARD domain located at the N-terminus of MAVS is required to form active MAVS aggregates and is adequate in this regard. It is linked to MDA5 and RIG-first I's CARD domains (13). Interspecies genetic diversity study illuminates the broad evolutionary history of genes. It can be used to identify protein sites or domains that interact directly with viral components or provide an explanation for antiviral specificity (14). When a host interacts with a virus, a dynamic arms race occurs. When viruses discover ways to overwhelm the host immune system, the host proteins responsible for pathogen recognition must change in order to avoid or restrict subsequent infections. These mechanisms result in adaptation and counter-adaptation on the side of the host pathogen interaction, culminating in rapid co-evolution of both parties. This fast development on the molecular level is typically reflected in host defense genes, which provide strong evidence of continuous diversifying selection (15). Given the wide variety of serious and fatal diseases caused by viruses and the critical function of RLRs in the mammalian innate immune system, it was expected that the RIG-I, MDA5, and LGP2 genes would have been exposed to strong selective pressures in all mammals. TLRs, another family of mammalian PRRs, have previously been demonstrated to exhibit extraordinary evidence of positive genetic selection in response to pathogen-induced selective pressures (16).

Therefore, we sought to determine whether MAVS exhibits evidence of being subjected to positive selection, as has been demonstrated for other duplicated genes, and whether this signal appears genuine or whether it could be an artifact of sequence level constraints due to the unusual genic organization of the gene family in question. Following sequence level studies to uncover potential positive selection residues, we looked at the structural and functional features of the detected sequence alterations to better understand their biological consequences.

## Materials and Methods

The nucleotide and amino acid sequences of MAVS proteins from mammalian species were obtained from the NCBI and KEGG databases (17). They included any protein for which there is direct molecular evidence of an immune role in mammalian species. The nucleotide and amino acid sequences of MAVS proteins from mammalian species were obtained from the NCBI and KEGG databases (Supplementary Table) (18). The maximum likelihood technique was used in the phylogenetic analysis carried out in MEGA6. In the bootstrap test, taxa were grouped using the maximum likelihood technique on 1,000 repetitions, and the results were analyzed (19, 20).

### Sequence Analysis

The sequences were modified and organized using the BioEdit program (21). After individual sequences were aligned, CLUSTALW was used to align different species' sequences together (22). It was necessary to confirm sequences similar to previously published sequences from passerine species using the NCBI BLAST program (23). ClustalW was used to match the sequences of each gene individually using the outgroup taxon *Myotis davidii* and the default automated alignment option. Each consecutive alignment was utilized for MEGA 6's Maximum Likelihood tree inference. A computer programme called MEGA6 was used to compute the average pairwise nucleotide distances (24) and the Poisson-corrected amino acid distances, which were utilized to calculate the distances between amino acids. We estimated standard errors by repeating the data 1,000 times using the bootstrap method (25).

### Inference of Recombination

We investigated recombination first because it can affect the outcomes of selection. In version 4 of the Recombination Detection Program, exon nucleotide alignment studies were implemented (RDP4) (26). Numerous techniques were employed to detect recombination events, including RDP (27), Chimera (28), BootScan (29). The Datamonkey webserver's online GARD tool (30) was utilized to analyze recombination signals. Recombination can provide a significant selective advantage, according to our findings. As allele frequencies at individual loci increase in response to selection, random recombination gradually pieces together chromosomes that contain increasing numbers of favorable alleles, resulting in a significant selective advantage (31).

### Tests for Selection

A variety of methods were used to identify which MAVS locations should be evaluated for selection. Using DnaSP 5.0 (32), it was estimated that the first standard selection test could be considered. For the overall dN/dS of MAVS codons, MEGA6 and the Nei-Gojobori approach (33) was utilized in conjunction with each other. Positive selection was detected in PAML 4.9 by using the maximum likelihood technique developed in codeml to find places that had been subjected to positive selection, as indicated by a ratio of (dN/dS) larger than one (34, 35). The models M7 (beta) and M8 were the two matched models that were investigated. To evaluate whether or not the alternative model (M8) provided a better fit than the M7, we used a distribution two to compare log-likelihood ratios (2lnL) to assess whether or not M8 offered a better fit than the M7. We used the Bayes empirical Bayes approach to find posterior probabilities in the M8 model, and the results were correct. In addition to the methods described above, positively selected sites were independently verified at each codon site using a variety of complementary methods carried out in datamonkey (http://www.datamonkey.org/) (30) using a range of complementary approaches MEME, FEL, SALC, and FUBER carried out in datamonkey (35, 36).

### Phylogenetic Analysis

We build two phylogenies using Bayesian inference (one for the sampled species and another for MAVS sequences from related passerines and sampled species). MrModeltest (37) and the Akaike Information Criterion (AICc) (38) were used to identify the best-fitting GTR+T nucleotide substitution model (39). Two million generations of Bayesian Markov chain Monte Carlo (MCMC) were run with 1,000 generations of sampling to determine whether log Likelihood entered the stationary phase (40). MrBayes v3.1.2 (41) was used to summarize the phylogenetic tree, with the top 25% removed. We utilized MEGA6's maximum likelihood (ML) approach to explore the connection between the sampled and related bird species (42). The data were examined using the T92+G model. Support was assessed using 1,000 bootstrap repeats. Values of more than 75% were seen in the ML phylogenetic trees. The second stage is to ascertain which amino acids have been selected affirmatively and whose existence is confirmed by the probability text. As a result of the Bayes theorem, we may deduce the posterior probability of each site from groups of sites of various kinds (43). Values greater than one are assigned to amino acid residues having a high chance of positive selection under range. It was necessary to test the aligned MAVS protein sequence in the Selecton version 2.2 server (http://selecton.tau.ac.il/), which allows for the different MAVS gene ratios to vary between codons within the aligned structure calculated using Bayesian inference method (34) by using different probability testing methods, to confirm positive codon selection. The Selecton's tests were also shown in a variety of colors, suggesting that they were subjected to a variety of various choosing procedures.

### Conservation Analyses

We examined the conserved synteny of genomic regions adjacent to the MAVS gene in mammalian species and discovered that it was pretty high in most of them. Using the ConSurf library, it was possible to assess the evolutionary distinctiveness of these genes by examining the evolutionary survival of human MAVS proteins across time (consurf.tau.ac.il/) (44), which was explicitly designed to study the evolutionary survival of human MAVS proteins. Changes in conserved amino acid sequences have a more significant negative impact on protein function and structure than polymorphisms in variable regions of the protein. Given that conserved synteny is associated with both gene function and expression (35), we performed an enrichment analysis to determine the biological importance of synteny genes by probing them in a variety of programs, including the enrichment analysis program the EnrichNet (45), to determine their biological significance. In addition to identifying genes in various molecular structures, EnrichNet can estimate the gaps between genes and pathways in a reference database (46) by employing an arbitrary selection technique.

### Protein Modeling and Structural Analysis

In this study, we used the Swiss model (http://swissmodel.expasy.org) online tool (47), I-TESSAR (48), and Phyre2 (http://www.sbg.bio.ic.ac.uk/phyre2/html/page.cgi?id=index) (49) to build the crystal structures of the human MAVS protein. In this study, the structure of proteins was predicted using a technique known as homology modeling. The assembled target proteins were minimized in UCSF Chimera 1.10.1 using the Amber force field and the conjugate gradient technique developed by the University of California San Francisco. Additionally, the ProSA webserver (50) was used to assess the stereo-chemical characteristics of the anticipated structures.

## Results

In this study, the mammalian MAVS have been examined to identify the adaptive selection and evolution in protein sequences. The MAVS protein has been identified as the major protein implicated in innate signaling against bacterial and fungal infections (51). Positive selection signals have been detected in these proteins, as demonstrated by a plethora of genetic markers, including higher non-synonymous exchange rates, large homologous haplotypes, and a lack of genetic diversity. As a result, as indicated by our findings, this signaling protein family has been under intense evolutionary pressure throughout its evolutionary history. We examined a set of 26 MAVS protein-coding orthologs shared by the human, monkey, dog, cat, cow, mouse, and domestic yak genomes to detect positive selection signals (Supplementary Table). In MAVS genes with significant signals (*P* < 0.05 corrected), branch site analyses revealed evidence of positive selection along the mammalian lineage (Supplementary Table).

### Adaptive Evolution of MAVS Proteins

**Figure 1:**
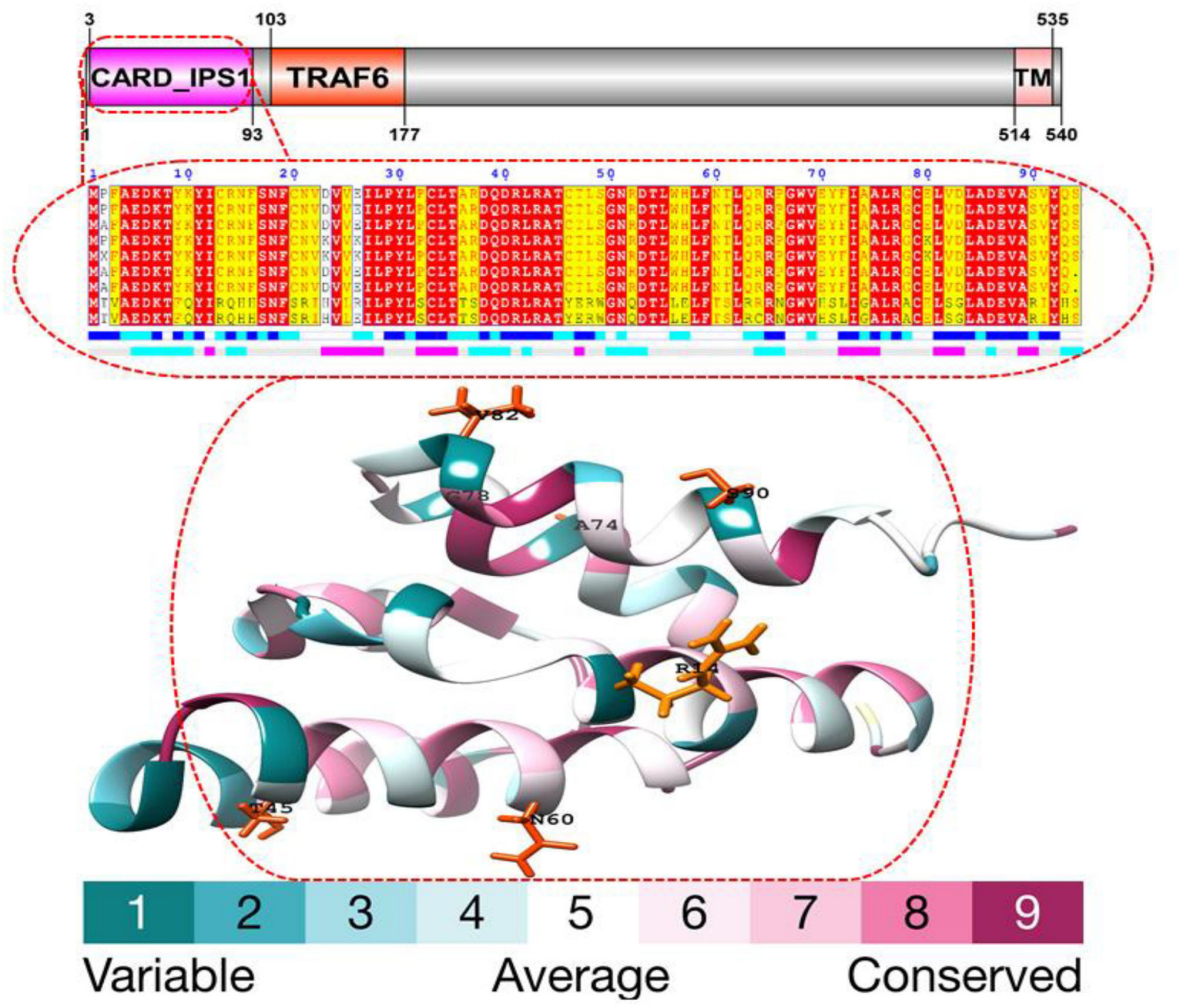
Analysis of the MAVS protein structure and conserved domains. The MSA of the 20 proteins with the highest homology to MAVS (as determined by a BLAST+ search against the PDBAA database). The accessibility of a solvent is shown by a first bar below the sequence (blue represents accessible, cyan represents intermediate, and white represents buried), and the hydropathy of a solvent is represented by a second bar below the sequence (pink is hydrophobic, white is neutral, cyan is hydrophilic). Residues that are the same or similar are boxed in yellow or red. The MAVS conserved CARD domain contains positively chosen amino acid residues. The crystal structure of human MAVS as a reference and positively selected sites were drawn onto the crystal structure using the Phyre tool.

**Figure 2:**
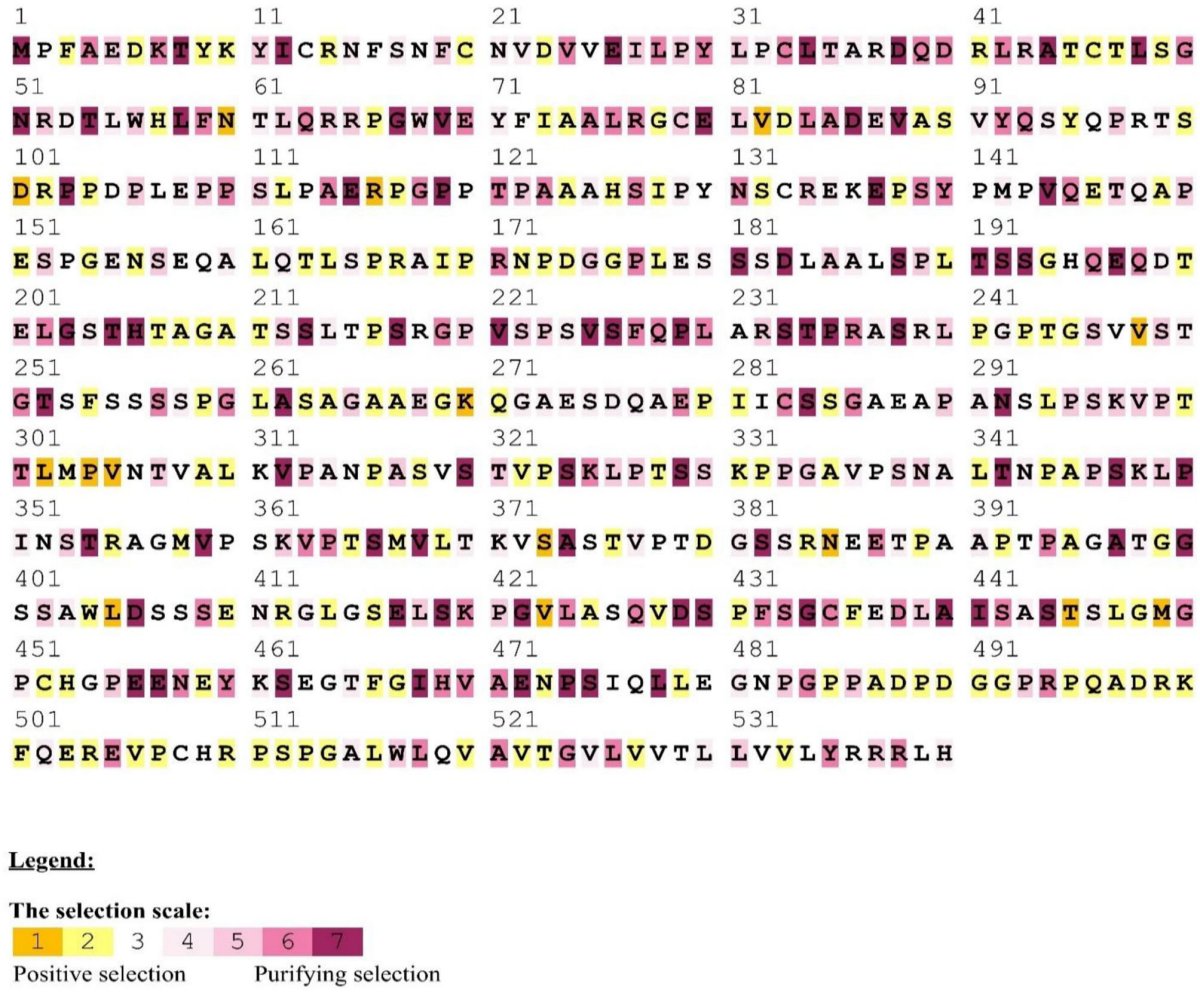
Positive/purifying selection was detected in MAVS homologous sequences from mammals using the human as a reference. Yellow and brown highlights represent positive selection, gray and white highlights describe the neutral selection, purple highlights on codons represent purifying selection.

Using various site models, we were able to identify genes that were under positive selection across mammalian species. We evaluated several models for the genes from the data set that were chosen. Probability analysis was used to evaluate alternative models based on a range of ratios to categorize the codons in the corresponding positively chosen genes. The results were shown in Table 1. The positive selection test was carried out with the help of two sets of models: M1a; M2a and M7; M8, respectively. In comparing M0 and M3, the likelihood test value was 2lnL = 114.827712 (*p* < 0.05). Still, the findings were significant in M1a vs. M2a, where the likelihood test value was 2lnL = 31.398468 (*p* < 0.05), and an M7 vs. M8, where the likelihood ratio test value was 2lnL = 32.374384 (*p* < 0.05) (Table 1). The HyPhy program was then used to investigate the positive selection evidence. MEME, FEL, and SLAC studies were performed to infer further positive selection signals. In this way, sites found using various strategies (and which were consistent with two or more approaches) were judged to be excellent candidates for positive selection. Our findings offered convincing evidence that natural selection had successfully selected these genes in mammals. By estimating the posterior probability for each codon, we identified the sites under selective strain using the Bayesian method. Sites having a greater probability of occurrence than sites with a lower probability of occurrence are more likely to experience positive selection with either >1 (Figure 1). Using BEB analysis, we were able to identify numerous positive selection sites in these proteins, with the bulk of these sites having a high retrospective probability of 95%. We observed that many sites were identified as under selection pressure throughout evolution, which further validated the positive consequences of selection (Figure 2). The Selecton technique can anticipate the adaptive selection pressure applied at certain codons. We utilized the ConSurf server (52) to forecast nucleic acid locations and the degree of conservation of amino acids in these two genes across different mammalian species (Figure 2). In our study, we discovered that the majority of locations that were favorably selected remained stable throughout the evolution of mammalian clades. The neural network algorithm residues of these proteins have been shown to have many conserved amino acids with a positive signal range, which can be found either visible or hidden.

**Table 1:** Likelihood Log ratios and PAML site models for positive selection.

**Gene**	***N***	***Lc***	***S***	**Model**	**Categories**	***2Δl*** ***M3 vs. M0***	***2Δl M2 vs. M1***	***2Δl*** ***M8 vs. M7***	**Parameter estimates**	**Positively selected sites**
*MAVS*	45	560	2.27	*M1*	Nearly Neutral (2 categories)	114.827712	31.398468	32.374384	*P*1 = 0.34017; *P*2 = 0.65983	14, 45*, 60*, 74, 78, 82*, 90*, 116*, 126, 149**, 163*, 312**, 438**, 465*, 513*
									ω1 = 0.06983; ω2 = 1.00000	
				*M2*	Positive Selection (3 categories)				P1 = 0.33817; P2 = 0.57824; P3 = 0.08360	
									ω1 = 0.09769; ω2 = 1.00000; ω3 = 3.81373	
				*M3*	Discrete (3 categories)				P1 = 0.23466; P2 = 0.65644; P3 = 0.10890	
									ω1 = 0.00868; ω2 = 0.82550; ω3 = 3.45775	
				*M7*	Beta (10 categories)				*P* = 0.04076; *q* = 0.01519	
				*M8*	Betaandw>1 (11 categories)				*P*0 = 0.90653; *p* = 0.23986; *q* = 0.13299	
									*p*1 = 0.09347; ω1 = 3.64677	

*The beta distribution parameters p and q, and the fraction of sites under positive selection (p1). Positive selection parameters are bolded. p: significant at 5%; p: significant at 1%. Positive selection sites found by model M8 are listed by human sequence numbering. Sites with high posterior probability. 0.8–0.9 in bold, and 0.5–0.7 in normal print. In this case, the critical values are 5.99, 9.21, and 13.82, respectively, for a l distribution with 2 degrees of freedom*.

** *significant at 1%*;

* *significant at 5%*.

We found an average of 22 MAVS protein locations subjected to positive selection. In all, we found 22 conserved sites under positive selection in the CARD-IPS1 domain. To eliminate PAML false positives, we employed a Selecton server (53) that detects adaptive selection at a specific amino acid position in the protein using the Mechanistic Empirical Combined Model (MEC). The MEC model quantifies variations in amino acid substitution rates. As a result, we discovered adaptive selection in the MAVS protein (Figure 2). Our research found that these proteins include Ig-V-like domains that have been implicated in several places. Positively chosen proteins may be retained and subjected to purifying selection throughout adaptive evolution.

**Figure 3:**
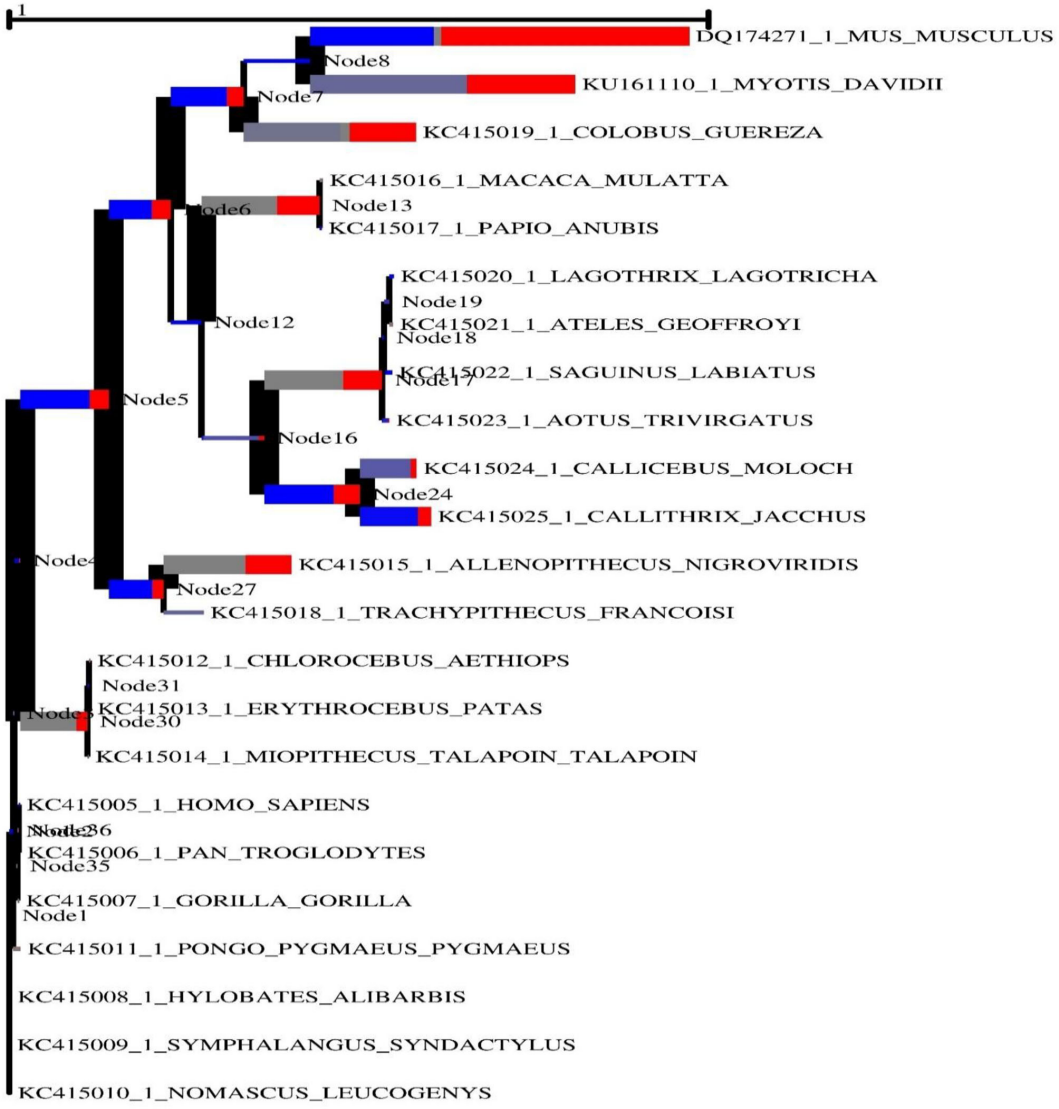
The adaptive branch-site REL test for episodic diversifying selection in the MAVS gene across vertebrate species is a new approach to testing for episodic diversifying selection. To calculate the size of the phylogenetic tree, the predicted number of substitutions/nucleotides was considered. With primary red corresponding ω> 5, primary blue corresponding ω= 0, and corresponding gray ω= 1, the hue of each color correlates to the degree of selection made for that color.

In order to generate positive selection signals, a lineage specific selection strategy must be used in conjunction with certain codons that are under selection pressure in a number of distinct lineages. In taxonomy and biological function prediction, biochemical sequences are frequently used to infer phylogenies from which to infer phylogenies. The process of creating a phylogeny often entails matching sequences. We employed an adaptive branch-site random effects likelihood (aBS-REL) model to quantify selection probability and identify lineage-specific selection for each phylogenetic grouping to find lineage-specific selection. This paradigm was developed at the University of California, Berkeley, and is credited with its invention. Following that, each gene was evaluated using the aBS-REL program in order to identify lineages that had undergone positive selection over the course of evolutionary adaptation. The aBS-REL model indicated that the genes identified by BUSTED as being under positive selection in mammalian lineages were also under positive selection, demonstrating that the two models were complementary, as evidenced by our findings (Figure 3). The branch-site-REL (BSR) program, which runs on the Data Monkey Web Server and searches for clades with statistically significant positive selection signals (*p* < *0.05*) in the avian, mammalian, and reptile lineages, discovered clades with statistically significant positive selection signals (*p* < *0.05*) in the avian, mammalian, and reptile lineages.

**Figure 4:**
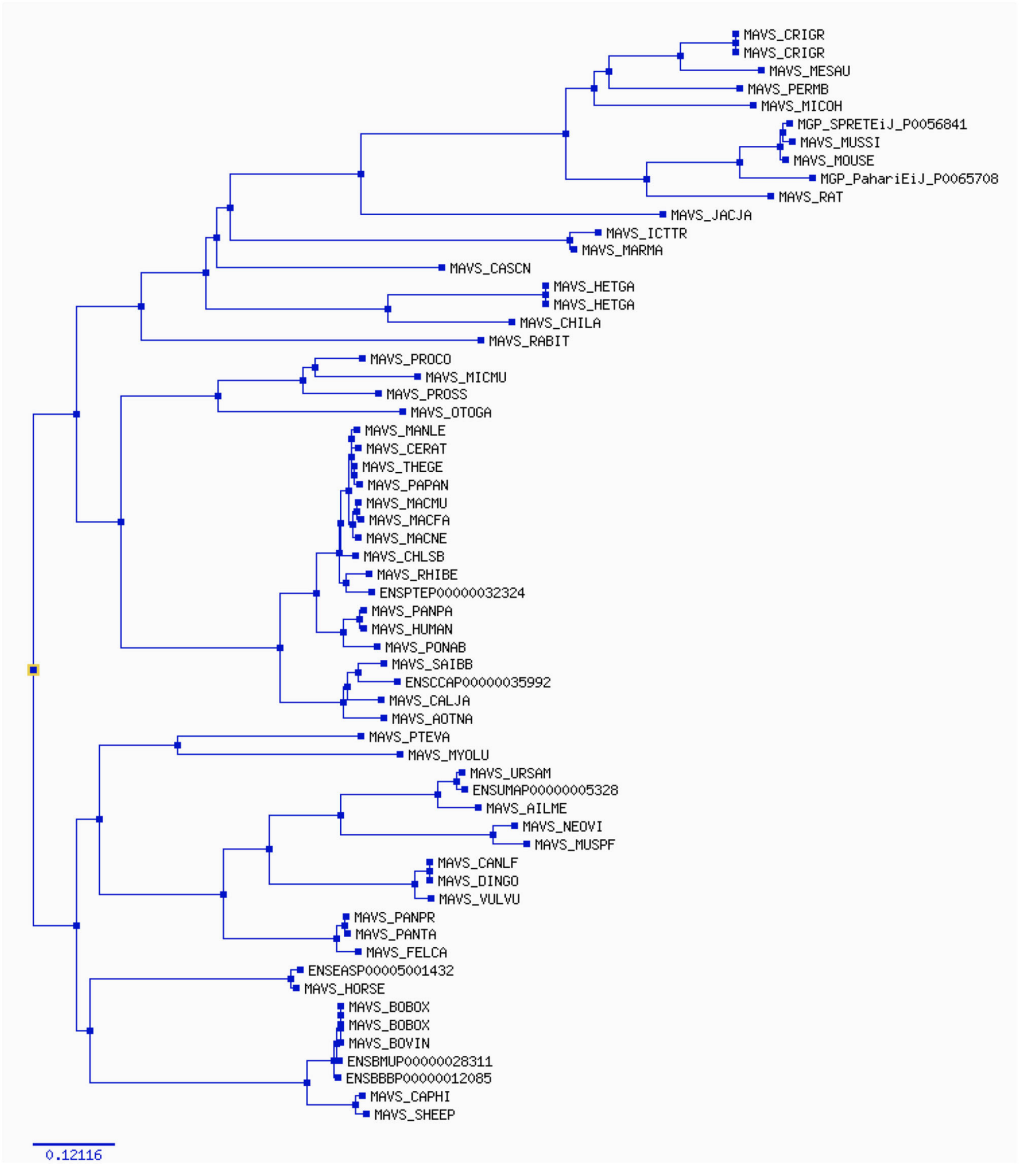
The phylogenetic tree estimates various parameters (*dN/dS*, branch length) on the codon (nucleotide) alignment for MAVS gene in mammalian species. The rate variation among codons was estimated using ω = *dN/dS*, which indicates selective pressure in Selectome database, to account for selection at the DNA level. Selectome uses the branch-site model, which estimates different dN/dS values among branches and sites.

The width of each color component corresponds to the percentage of sites that fall into the associated class in the classification hierarchy. Using a corrected 0.05, the sequential likelihood ratio test revealed that larger branches had undergone episodic diversifying selection, but thinner branches had not. The Selectome database (https://selectome.unil.ch/) was used to undertake an evolutionary analysis of positive selection. By comparingessentially neutral evolutionary models, we were able to calculate the fraction of preferentially selected gene sites in genes across mammalian species. Non-synonymous substitution rates (*dN/dS*) were greater at the chosen sites than expected when the parameter was set to one. Selectome accepts gene requests for both positive selection outcomes and gene-related principles, which may be seen on the Selectome database. The positively picked findings may be seen in the tree and matching protein sequences. When a gene was selected by Darwinian selection, it was selected for a specific subset of sites in the phylogenetic tree. The Selectome server identified species branching in MAVS genes, which the researchers confirmed. Positive selection phylogenetic branching (site test) were determined in just a few cases overall (Figure 4).

## Discussion

We examined a set of 26 MAVS protein-coding orthologs shared by the human, monkey, dog, cat, cow, mouse, and domestic yak genomes to detect positive selection signals (Supplementary Table). In MAVS genes with significant signals (*P* < 0.05 corrected), branch site analyses revealed evidence of positive selection along the mammalian lineage (Supplementary Table). Several evolutionary processes occur in animal genomes, including divergence, integration of genetic material from lineages, duplication events, and epigenesis. Divergence is one of the most well-studied evolutionary processes. On a horizontal scale, genetic transfers and duplications serve as the building blocks for developing all major adaptive immune molecular systems (54). Previously, studies on the evolution of immune genes in birds focused on the co-evolution of disease hotspots such as MAVS in influenza virus infection (Mammalian Apicomplexan Viral System) (55). They contribute to lymphocyte activation, immune system activity, T regulatory cell stimulation, and the origin, development, and immunological tolerance of autoimmunity (56). MAVS genes have been identified to exhibit positive selection in mammals (Table 1). We observed that the genes with the greatest overall characteristic expression in these mammalian lineages give standards similar to those reported in animals that were not chosen or were positively selected in the laboratory. Our findings suggest that pathogens are a continuous selective constraint throughout the vertebrate phylogenetic tree. According to previous research, an adaptive immune system was close to the first mammalian evolutionary transition of MAVS (Mammals Adapting to Variable Environments) (36). In the presence of viral antagonism, host antiviral factors such as MAVS can be subjected to consistent selective pressure, resulting in positive selection (i.e., accumulation of an excess of nonsynonymous changes relative to synonymous changes over evolutionary time). In the previous studies, positive selection has been shown in numerous antiviral factors that have been identified in primate genomes (57). It is anticipated that positive selection is mostly driven by adaptations to previous viral infections, with adaptive modifications having advantageous repercussions for the host to overcome viral antagonism. However, the adaptive alterations that occur from this process can potentially impact resistance or susceptibility to modern viruses. In the case of the antiviral gene Protein Kinase R (PKR), adaptive modifications at critical residues in the gene are essential drivers of PKR's capacity to withstand antagonism by modern poxviruses (57, 58), which are likely driven by ancient viruses. As a result, antiviral genes that have evolved through positive selection are excellent candidates to serve as genetic determinants of resistance or susceptibility to emerging viruses. It is thought that the MAVS family has been split into two branches, both of which are found in all cnidarians and bilaterians, according to current knowledge (55). A positive selection evolutionary model (M8) was used to identify the difference at the codon level. An MCMC model, employed in MrBayes on the Selecton server, was used to determine the difference at the codon level (59). In both situations, values for each place were calculated. Our findings show Ig domain conservation in MAVS coding sequences following MAFFT protein alignments (Figure 2). These findings show that non-identical protein switches in purifying Selection areas are harmful to health and hence unlikely to be fixed during evolution (20, 40). Next, positive selection selected amino acid residues with ω > 1 (Table 1). Comparing amino acids N60 and A74, G78 and V82 in CARD domain (Figure 2). Using the M8 evolutionary model, three positively chosen sites, N60 and A74, G78 and V82, were identified in MAVS, with a *dN/dS* ratio of 10.89870. Amino acid sites in other proteins under strong positive selection have developed faster than mature ones (20). The dynamic selection causes the modification to enhance protein secretion effectiveness, which is true for MAVS, unlike matured protein (60, 61). According to the research, MAVS from several primates were shown to be resistant to inactivation by the HCV protease. A single alteration inside the protease cleavage region in MAVS has been identified as the source of this resistance. These modifications prevent MAVS from being cleaved by the HCV protease. Surprisingly, most of these modifications have occurred independently at a single residue 506, which has developed under positive selection throughout time (57). In our study we have detected.

We detected positive selection in mammalian and avian clades in the MAVS gene (Figure 3). Interestingly, we detected positive selection in most mammalian clades in the data set when looking at the MAVS gene. Given that multi-nucleotide mutations might lead to erroneous positive selection implications in branch-site analysis, we further verified our results using the aBSREL (42). Similar selection patterns were identified between the aBSREL and site-model (Figure 3). In general, the MAVS protein found little evidence of positive selection in vertebrate lineages, except in the avian group. The LRT fully supports the M8 evolutionary model's observation of no positive selection. This may be related to the absence of gene duplication events in the evolutionary history of the MAVS gene. Gene duplication is one of the evolutionary strategies that allow genomes to evolve. Positive selection occurs following a duplication event for other proteins, suggesting a relaxation of the selective pressure supporting genetic diversity (35, 62). Positively chosen sites are found in the helicase domain. The majority of the positively chosen sites in MAVS are in areas exclusive to this RLR, including a unique insertion inside the helicase domain. According to structural analysis, positively selected residues are implicated in para-influenza virus protein binding. Our findings suggest that RLRs have been involved in host-virus genetic conflict, leading to diversifying selection, and that they have shown parallel evolution at the same place in RIG-I, a position that is likely to be critical in antiviral responses.

Furthermore, among 22 mammalian species, we found strong selection signals in the hominidae clade, which resulted in 149A, 438V, and 465M codon positions. Positively chosen amino acid sites are critical for signaling. These receptors detect viral RNA and activate signal transduction *via* contact with the adaptor protein MAVS, culminating in the generation of pro-inflammatory cytokines and type I interferon.

Not just in birds but also in other vertebrate lineages throughout MAVS evolution, giving Bayesian phylogenetic methods (Figure 4). These findings suggest that negative selection has largely dictated PD1 atomic progress. Variations in a few motifs discovered in MAVS may be illustrated by altering the genetic basis for homologous arrangements in mammals and other animals. Purifying choice, notably inside areas compared to Ig similar domain, has pushed MAVS molecular evolution. During MAVS evolution, residues within the areas compared to each motif co-vary with each other (63, 64).

Protein structure and function rely on coordinated amino acid interactions. Thus, identifying structural features of favorably chosen amino acid residues might be done by detecting co-evolving sites. Their physical or functional connection may have resulted in this co-evolutionary relationship. Protein co-evolution has been linked to protein stability and intermolecular interactions (34, 40). Thus, evolution may have changed MAVS proteins. By calculating the *dN/dS* of mammalian MAVS sequences, we observed that N60 and A74, G78 and V82 of the human CARD domain might be relevant for certain earlier suggested activities. Its domains are well conserved among vertebrates, indicating that their functions are not redundant and that the selection pressure may come from MAVS specialization for immune system control. Using protein alignments for MAVS homologs as input, we discovered positively chosen co-evolving residues with considerable heterogeneity (20). These findings suggest that domain-like areas represent structural and functional components within these proteins. Using a Gaussian network, homology modeling the region similar to CARD domains. According to its projected molecular mass of 56KDa, MAVS is a mitochondrial integral outer-membrane protein that in healthy conditions forms a supramolecular complex (about 600KDa) (65). MAVS is encoded in the nuclear genome (not mtDNA) and is widely expressed in tissues and cells. Some MAVS orthologs are conserved across fish species (66). MAVS has an amino-terminal caspase activation and recruitment domain (CARD) with six helices, three of which create a flat positively charged surface and two of which form an acidic negatively charged surface (4).

## Conclusion

Detecting evolutionary arms races is critical for comprehending and controlling host-pathogen interactions, such as viral competition with their animal hosts. The need for innate immune activation for cellular energy output and significant metabolic reprogramming has necessitated mitochondrial integration into this arm of immunity, which has evolved to develop and increase the cross-talk between metabolism and innate immune pathways. Arms races may be identified at the molecular level by detecting rapidly changing nucleotide sequences. The purpose of this study was to determine whether or not sequence-level analyses are an adequate method for detecting what is ultimately a phenotype-level effect. These findings shed light on the adaptive development of mitochondrial antiviral proteins in mammalian species. The CARD domain in MAVS proteins are highly conserved in mammals, which may help explain its involvement in biological systems, including adaptive immunity. Due to the vast data collection and recommendations from different study areas, the evolutionary history of MAVS protein is revealed. We discovered that the evolutionary rates of mammalian and other vertebrate lineages differ. Further research into the functional ramifications of positive selection signals at the cellular and organismal levels would help distinguish between convergence and early shared vertebrate selection. Finally, the inclusion of evolutionary diversity may contribute to clarifying some of the conflicts that have developed in mammalian proteins due to a variety of experimental data. Following these findings, we propose that studies of the co-evolution of rapidly evolving immune proteins may give data compatible with the interaction between the host and pathogens.

## Data Availability Statement

The original contributions presented in the study are included in the article/Supplementary Material, further inquiries can be directed to the corresponding author.

## Author Contributions

HIA, GA, and MKM: data curation. HIA, GA, and BS: formal analysis and methodology. JC: funding acquisition and supervision. JC and MKM: investigation and project administration. HIA and JC: resources and visualization. HIA, MNI, and MAI: software. MKM and BS: validation. HIA and GA: writing and original draft. HIA, GA, MKM, MNI, and MAI: writing and review and editing. All authors contributed to the article and approved the submitted version.

## Funding

This work was supported by the GDAS project of Science and Technology Development (2019-GDASYL-0103059 and 2018GDASCX-0107).

## Conflict of Interest

The authors declare that the research was conducted in the absence of any commercial or financial relationships that could be construed as a potential conflict of interest.

## Publisher's Note

All claims expressed in this article are solely those of the authors and do not necessarily represent those of their affiliated organizations, or those of the publisher, the editors and the reviewers. Any product that may be evaluated in this article, or claim that may be made by its manufacturer, is not guaranteed or endorsed by the publisher.
