# Supplementary material for: Positive Selection Drives the Adaptive Evolution of Mitochondrial Antiviral Signaling (MAVS) Proteins-Mediating Innate Immunity in Mammals
Source: Front Vet Sci. 2022 Jan 31;8:814765. doi: 10.3389/fvets.2021.814765 (PMC8841730; doi:10.3389/fvets.2021.814765)
Supplement: Supplementary file 1 [file Table_1.docx]

**Supplementary table: Genbank accession numbers of mammalian species used for sequence analysis.**

| **Species Name** | **Scientific Name** | **Genbank Accession Number** | **Ensembl Accession Number** |
| --- | --- | --- | --- |
| Human | *Homo sapiens* | KC415005.1 | ENSG00000088888 |
| Monkey | *Macaca mulatta* | KC415016.1 | ENSMMUG00000017150 |
| Western gorilla | *Gorilla gorilla* | KC415007.1 | ENSGGOG00000010178 |
| Olive baboon | *Papio anubis* | KC415017.1 | ENSPANG00000003902 |
| Chimpanzee | *Pan troglodytes* | KC415006.1 | ENSPTRG00000013209 |
| White cheeked Gibbon | *Hylobates alibarbis* | KC415008.1 | ENSNLEG00000007664 |
| Cattle | *Bos taurus* | XM_025000837.1 | ENSBTAG00000013545 |
| Buffalo | *Bubalus bubalis* | XM_006072927.4 |  |
| Sheep | *Ovis aries* | XM_004014360.5 | ENSOARG00020003796 |
| Goat | *Capra hircus* | MT501722.1 | ENSCHIG00000015699 |
| House mouse | *Mus musculus* | XM_036161358.1 | ENSMUSG00000037523 |
| Norway Rat | *Rattus norvegicus* | XM_006235035.4 | ENSRNOG00000025295 |
| Rabbit | *Oryctolagus cuniculus* | XM_002710888.3 | ENSOCUG00000012269 |
| Dog | *Canis lupus familiaris* | XM_038432825.1 | ENSCAFG00000006157 |
| Cat | *Felis catus* | XM_006929884.5 | ENSFCAG00000022983 |
| Bat | *Myotis davidii* | KU161110.1 |  |
| Marmoset | *Callithrix jacchus* | KC415025.1 |  |
| Pig | *Sus scrofa* | MK302496 | ENSSSCG00070022209 |
| American bison | *Bison bison* | XM_010836470.1 | ENSBBBG00000019965 |
| Arabian camel | *Camelus dromedarius* | XM_010988239.2 | ENSCDRG00005009767 |
| Chinese hamster | *Cricetulus griseus* | XM_027421749.2 | ENSCGRG00001023482 |
| Wild yak | *Bos mutus* | XM_014477646.1 | ENSBMUG00000021216 |
| Tiger | *Panthera tigris* | XM_007073223 | ENSPTIG00000010949 |
| Tree shrew | *Tupaia belangeri* | KM005100.1 |  |
| Fruit bat | *Eidolon helvum* | MK096783 |  |
| Bank vole | *Myodes glareolus* | MK096788 |  |
